# Time–frequency analysis of serum with proton nuclear magnetic resonance for diagnosis of pancreatic cancer

**DOI:** 10.1038/s41598-020-79087-3

**Published:** 2020-12-14

**Authors:** Asahi Sato, Toshihiko Masui, Akitada Yogo, Takashi Ito, Keiko Hirakawa, Yoshimasa Kanawaku, Kaoru Koike, Shinji Uemoto

**Affiliations:** 1 Division of Hepato-Biliary-Pancreatic Surgery and Transplantation, Department of Surgery, Postgraduate School of Medicine, Kyoto University, 54 Shogoin-kawaharacho, Sakyo-ku, Kyoto, Japan; 2 Department of Surgery, Shiga General Hospital, Shiga, Japan; 3 Research Laboratory for Magnetic Resonance, Nippon Medical School, Tokyo, Japan; 4 Department of Legal Medicine, Nippon Medical School, Tokyo, Japan; 5 Department of Primary Care and Emergency Medicine, Postgraduate School of Medicine, Kyoto University, Kyoto, Japan

**Keywords:** Diagnostic markers, Cancer, Biomarkers

## Abstract

Although serum markers such as carcinoembryonic antigen (CEA) and carbohydrate antigen (CA19-9) have been widely used in screening for pancreatic cancer (PC), their sensitivity and specificity are unsatisfactory. Recently, a novel tool of analyzing serum using the short-time Fourier transform (STFT) of free induction decays (FIDs) obtained by ^1^H-NMR has been introduced. We for the first time evaluated the utility of this technology as a diagnostic tool for PC. Serum was obtained from PC patients before starting any treatments. Samples taken from individuals with benign diseases or donors for liver transplantation were obtained as controls. Serum samples from both groups underwent ^1^H-NMR and STFT of FIDs. STFT data were analyzed by partial least squares discriminant analysis (PLS-DA) to clarify whether differences were apparent between groups. As a result, PLS-DA score plots indicated that STFT of FIDs enabled effective classification of groups with and without PC. Additionally, in a subgroup of PC, long-term survivors (≥ 2 years) could be discriminated from short-term survivors (< 2 years), regardless of pathologic stage or CEA or CA19-9 levels. In conclusion, STFT of FIDs obtained from ^1^H-NMR have a potential to be a diagnostic and prognostic tool of PC.

## Introduction

Pancreatic cancer (PC) is the fourth leading cause of cancer-related death in Western societies and is predicted to become the second leading cause by 2030 in the United States^1–3^. Although significant efforts to uncover the basic pathophysiology and identify effective treatments of PC have been made, improving the prognosis of PC remains challenging^4–6^. Surgery is the only potentially curative option, but only approximately 20% of patients are eligible for surgical resection^1,2^. Diagnosis of PC at a less-aggressive stage is considered likely to facilitate improved cure rates by way of surgical resection. Several investigators have thus studied the utility of laboratory-determined markers such as carbohydrate antigen 19-9 (CA19-9) and carcinoembryonic antigen (CEA)^7–12^. However, rates of early and specific detection of PC using serum markers have not been satisfactory because tumor markers are not always elevated in the serum of patients with PC, and their levels can be affected by diabetes, smoking, and obstructive jaundice^13,14^.

If patients with PC have particular disease-specific gene products in serum, diagnosis of the disease by measuring levels of these products might be feasible. Indeed, PCs contain products from accumulated genetic mutations, resulting in a variety of proteomic and metabolomic changes^4,15–18^. That is, the variety of mutations is likely so wide that establishing a measuring system for every product would be impractical. Some investigators have therefore tried to apply a more comprehensive approach^16–18^. For example, Ludwig et al. identified 121 relevant serologic biomarkers by applying three complementary mass spectrometry approaches in a murine model of PC^18^. However, the results seemed to be nonspecific because only 38% of biomarkers they detected were relevant to PC according to the previous reports.

Nuclear magnetic resonance (NMR) is a phenomenon that occurs when the nuclei of certain atoms are moved from a static magnetic field to an oscillating magnetic field^19,20^. Radiofrequency pulse at the resonance frequency causes impulse responses so called free induction decays (FIDs) during NMR measurements. By analyzing FIDs using Fourier transform (FT), we can obtain characteristic spectrums of substances (frequency analysis). Some investigators have studied the utility of this modality in discriminating serum taken from patients with cancers such as renal cell carcinoma, hepatocellular carcinoma and lung cancer^21–23^. However, the time-dependent transition of the signal is lost during the process of analysis in principle. Short-time Fourier transform (STFT) is a basic method for time–frequency analyses^24–27^. We have recently reported that the STFT of FIDs obtained from proton NMR (^1^H-NMR) measurements offers a useful serological diagnostic tool^28^. No previous reports appear to have applied this analysis as a diagnostic tool for diseases, including PC.

In this study, we demonstrated the utility of the STFT of FIDs obtained from serum in distinguishing patients with and without PC. Furthermore, we tried to apply this method in separating PC patients with prognosis.

## Results

### Comparison between control and PC groups

Characteristics of the control and PC groups are described in Table 1. Age was significantly lower in the control group relative to the PC group (median 54 years vs. 73 years, *P* < 0.001). The proportion of females did not differ significantly between the control group (55%) and PC group (33%; *P* = 0.089). The control group consisted of 6 donors for liver transplantation, 9 patients with gallbladder stones, and 5 patients with inguinal hernia. The PC group included 9 patients (20%) with stage IV disease who were not indicated for surgery at the time of blood sample collection, whereas serum was obtained from 37 patients (80%) before curative surgery. With respect to known serum markers, the ratio of patients with a CA19-9 level above the cut-off (≥ 37 U/mL) was significantly higher in the PC group (65%) compared with the control group (5%; *P* < 0.001), whereas no significant difference was seen between groups with respect to CEA level above the cut-off (≥ 5.0 ng/μL) (10% vs. 30%; *P* = 0.137). The sensitivities of CEA and CA19-9 as a diagnostic marker for PC in our dataset were 30% and 65%, respectively, whereas specificities were 90% and 95%, respectively. The positive predictive values (PPVs) of CEA and CA19-9 were 88% and 97%, whereas negative predictive values (NPVs) were 36% and 54%, respectively.

**Table 1:** Characteristics of patients enrolled in our study.

	Control (n = 20)	PC (n = 46)	*P*
Age, years median (range)	54 (24–81)	73 (44–87)	< 0.001*
Female (%)	11 (55)	15 (33)	0.089
**Condition**			< 0.001*
PC	–	46	
Donor	6	–	
GBS	9	–	
Hernia	5	–	
**Clinical stage**			–
IA	–	1	
IB	–	2	
IIA	–	12	
IIB	–	17	
III	–	0	
IV	–	14	
CEA > 5.0 ng/mL (%)	2 (10)	14 (30)	0.137
CA19-9 > 37.0 U/mL (%)	1 (5)	30 (65)	< 0.001*

*PC* pancreatic cancer, *GBS* gallbladder stones, *CEA* carcinoembryonic antigen, *CA19-9* carbohydrate antigen 19-9.

**Figure 1:**
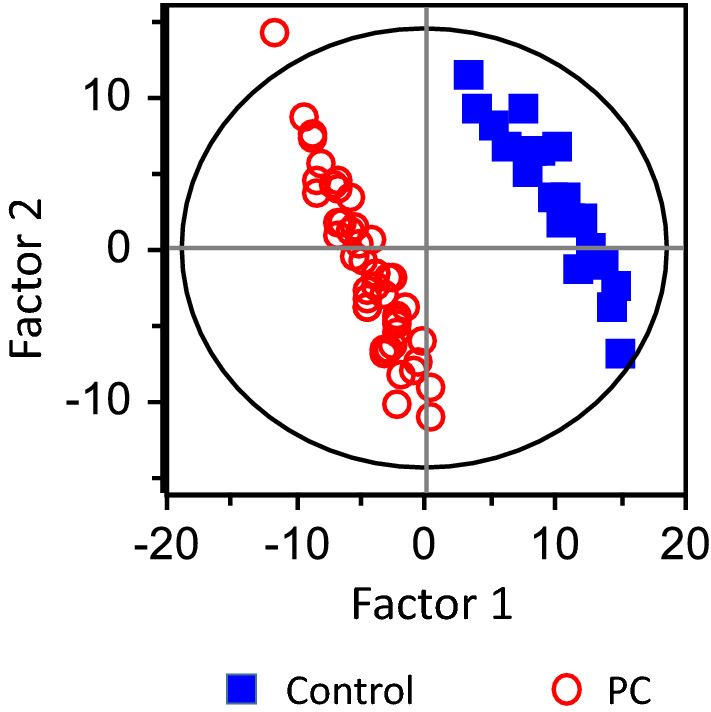
Plot prepared using FID STFT data comparing controls and patients with pancreatic cancer (PC). Blue closed squares represent controls and red open circles represent patients with PC (*R*^2^ = 0.989 and *Q*^2^ = 0.948).

A plot of PLS-DA scores is shown in Fig. 1. Each dot represents an individual sample. Factors 1 and 2 were calculated by PLS-DA algorithm using all 66 sets of ^1^H-NMR STFT data. Clear separation of the control and PC groups was observed in the PLS-DA scores plot (*R*^2^ = 0.989, *Q*^2^ = 0.948).

### Comparison between long- and short-term PC survivors

Patients with PC were categorized into long-term survivor (LS) and short-term survivor (SS) groups. Because median survival time for these patients was 24 months, we categorized patients who survived over 2 years after the examination as LS. No differences in demographic characteristics or pathologic staging were evident between the LS and SS groups (Table 2). When LS and SS patients were compared with respect to CEA and CA19-9 cut-off levels, no difference was observed between the two groups (CEA, LS vs. SS: 23% vs. 38%, *P* = 0.274; CA19-9, LS vs. SS: 55% vs. 75%, *P* = 0.144). Sensitivities of CEA and CA19-9 as prognostic markers for PC were 38% and 75%, respectively, whereas specificities were 77% and 45%, respectively. The positive predictive values (PPVs) of CEA and CA19-9 were 64% and 60%, whereas negative predictive values (NPVs) were 53% and 63%, respectively.

**Table 2:** Comparison of characteristics between long-term and short-term survivors after blood sample collection.

	LS (n = 22)	SS (n = 24)	*P*
Age, years median (range)	75 (49–87)	72 (44–82)	0.271
Female (%)	8 (36)	7 (29)	0.603
**Clinical stage**			0.183
IA	0	1	
IB	1	1	
IIA	9	3	
IIB	6	11	
III	0	0	
IV	6	8	
CEA > 5.0 (%)	5 (23)	9 (38)	0.274
CA19-9 > 37.0 (%)	12 (55)	18 (75)	0.144

*PC* Pancreatic cancer, *LS* long-term survivor, *SS* short-term survivor, *CEA* carcinoembryonic antigen, *CA19-9* carbohydrate antigen 19-9.

**Figure 2:**
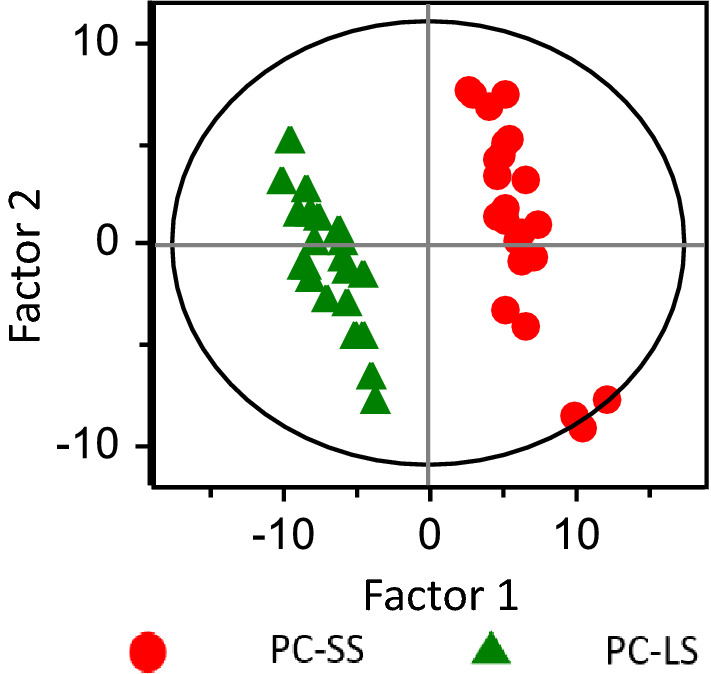
Plot prepared using FID STFT data obtained from patients with pancreatic cancer comparing long-term survivors (LS) and short-term survivors (SS). Green triangles represent patients surviving over 2 years (LS) and red closed circles represent patients surviving less than 2 years (SS) (*R*^2^ = 0.989 and *Q*^2^ = 0.914).

A plot of PLS-DA scores acquired using the STFT data is shown in Fig. 2. Each dot represents an individual sample. Factors 1 and 2 were calculated by PLS-DA algorithm using all 46 sets of ^1^H-NMR STFT data. Good discrimination between the LS and SS groups was observed with the PLS-DA scores plot (*R*^2^ = 0.989, *Q*^2^ = 0.914).

## Discussion

The present study examined whether the STFT of FIDs obtained from serum samples was effective for diagnosing PC. First, this method distinctly discriminated between the control and PC populations. Second, this method enabled better stratification of prognosis for PC patients compared with the traditional markers of CA19-9 and CEA.

To date, considerable research has been conducted to identify biomarkers for PC. Those studies can be divided into two categories: one related to pathologic biomarkers and the other to serologic biomarkers. Pathology-related information obtained by immunohistochemistry has been studied as a means of stratifying PC prognosis. Marechal et al. reported that sonic hedgehog and Gli1 expression predict outcome^29^. Quadri et al. recently reported that the expression pattern of connector enhancer of the kinase suppressor of Ras-1 (CNKSR-1) correlates negatively with survival time^30^. Even though these studies were informative because the markers identified are based on actual characteristics of PC, assessments can only be performed after invasive tissue sampling and the judgements might be subjective. In addition, factors such as the methodology used for fixation can affect the results. In contrast, analyses of serologic markers such as CEA and CA19-9 have been broadly applied for the detection of PC as less-invasive diagnostic tools^7–12^. However, the sensitivity and specificity of these markers is insufficient, and their expressions can be affected by conditions other than PC. Thus, more sensitive and specific modalities are sorely needed. Although it might be difficult to simply compare our methods and CEA or CA19-9 by the value of sensitivity and specificity because of the nature of supervised dimensionality reduction of PLS-DA, clear discrimination was performed in our analysis as indicated in Figs. 1 and 2.

Recently, interest has been growing in comprehensive profiling approaches, such as proteomics and metabolomics^16–18^, as the progression of PC is known to involve numerous gene mutations that produce a variety of characteristic proteins and metabolites^14^. This kind of approach is supposed to be closer to the real nature of the cancer. However, in principle, we cannot analyze materials that we do not know and therefore cannot measure, because such analyses provide only retrospective information. The current results showed the potential for novel time–frequency analysis, which does not identify the substance in the serum individually but targets the physical characteristics of serum. In other words, this approach didn’t intend to identify or compare each substance. Thus, we didn’t perform adjustment of pH and the amount of DANTE presaturation by samples because divergence caused by these factors might reflect the existence of the disease itself.

Recently, Michálková et al. showed the utility of ^1^H-NMR metabolomics by comparing spectrums (frequency analysis) and elucidated upregulated substances in the serum from PC patients in a pilot study^31^. Three key differences are apparent between the present investigation and their study. First, we did not intend to determine which specific substances changed in PC, but rather aimed to characterize serum according to more physical features using STFT (time–frequency analysis). Second, we conducted our study in a relatively larger number of cases. Third, we succeeded in not only diagnosing PC, but also stratifying prognosis. We speculate that our method of time–frequency analysis contains more information that might help achieve better stratification of patients with PC.

^1^H-NMR analysis is a well-established analytical tool in the field of materials chemistry. Thus, with increasing interest in this approach, we believe that the cost reductions that would result from mass production of the instruments as well as centralization of measurements make application of this tool into the dairy practice worthy of consideration. We propose this tool can be deployed in at least three ways into the dairy practice worthy of consideration: First, it can be used as a screening tool for detecting PC in a medical examination. Second, this method can be expanded as a tool for predicting prognosis. Finally, it could be applied to distinguish PC according to the sensitivity of chemoradiotherapy.

This study has several limitations. First, because of the relatively small number of samples, the study had low statistical power. Second, the population in this study comprised patients admitted to the surgical department and did not include patients with PC at an early stage. Thus, the utility of this tool has not been investigated adequately. To verify the utility in diagnosing PC in the early stage, serum samples collected during ordinary medical check-ups from a large cohort need to be analyzed. Third, we could not exclude the possibility that the difference of PLS-DA score affected by the difference in age and sex between PC and control group. Finally, further investigation is needed to elucidate the detailed mechanisms by which the STFT of FIDs differentiate the characteristics of serum samples. Combination with various liquid biopsy samples, e.g., circulating tumor cells, circulating tumor DNA and exosomes, may improve the accuracy of PC diagnosis.

In conclusion, we have demonstrated that the STFT of FIDs obtained from serum can diagnose patients with PC. A validation study in a larger population is necessary to achieve global application of this technology in the future.

## Methods

### Patients

Patients with pathologically diagnosed PC who were treated in Kyoto University Hospital between August 2013 and March 2016 were enrolled in this study. Volunteers including patients with various benign diseases (e.g., gallbladder stones, inguinal hernia) or living liver transplantation donors at the same institution in the same period were also enrolled as controls. Patients with benign diseases were examined by fecal occult blood testing and computed tomography to confirm the absence of malignancies. Living liver transplantation donors were included because they had been adequately examined to rule out health problems that could exclude them from becoming a donor. All patients and volunteers were informed about the objectives of the study and provided written informed consent prior to enrolment.

Information regarding patient demographics, laboratory data such as CEA and CA19-9 levels, and prognoses was obtained from the medical records of patients. The PC staging system used in this study was the American Joint Committee on Cancer TNM system. This study was performed in accordance with the declaration of Helsinki revised in 2013 and approved by the ethics committee at Kyoto University (approval number: E1751).

### Serum samples

Blood samples were obtained from candidates on admission via routine blood examination before starting treatment for PC. Samples were centrifuged immediately (3000×*g*, 5 min), and the supernatants were dispensed into microtubes and preserved at − 80 °C until analysis.

### Acquisition of FIDs from serum samples and STFT for time–frequency analysis

Methods for ^1^H-NMR measurement and FID analysis using STFT are described elsewhere^28^. Briefly, each serum sample (200 μL) was mixed with 400 μL of deuterium oxide (^2^H_2_O) (ISOTEC, Sigma-Aldrich, St. Louis, MO, USA) and pipetted into a 5-mm (outer diameter) NMR tube (Wilmad-LabGlass, Vineland, NJ, USA) for NMR analysis. Solution-state NMR analyses were performed at a proton resonance frequency of 300 MHz (7.05 T) using an ECX NMR spectrometer (JEOL, Tokyo, Japan) interfaced with a TH5 probe (auto-tunable type) equipped with Delta NMR processing and control software (version 4.3.2; JEOL). FIDs were acquired using a single pulse with a 2.0-s relaxation delay between repeated pulse sequences and subsequently processed using LabVIEW 2015 software (version 15.0.1f1; National Instruments Corp., Austin, TX, USA). STFT was performed using the FID data and spectrograms were generated as a visual representation of the time–frequency analysis. Each spectrogram datum in the two-dimensional matrix was read from left to right and row by row and populated in a single row. In this study, each spectrogram datum was reshaped from a 257 × 512 two-dimensional matrix into a 1 × 131,584 single row. After combining the total rows into a separate 66 × 131,584 matrix, the resulting dataset was used to perform multivariate data analysis.

### Statistical analysis

Data calculated using STFT were imported into Unscrambler X software (version 10.5; Camo Software AS, Oslo, Norway) for a partial least-squares discriminant analysis (PLS-DA)^28^. All data were transformed using standard normal variates to remove scatter effects, by centering and scaling each individual variable. The wide Kernel PLS algorithm was used for factor calculation. Important variables were detected for the separation of sample groups according to the loading weights. Cross-validation was used to statistically evaluate and compare learning algorithms by randomly dividing the data into a software-specified optimal number of segments. To estimate the goodness of fit for future predictions using a defined number of factors, *R*^2^ and *Q*^2^ were calculated for the explained-variance plots for calibration and validation data sets, respectively^28^.

Differences in categorical variables were analyzed using the chi-square test or Fisher’s exact test, and differences in continuous valuables were analyzed using the Mann–Whitney *U* test. *P* < 0.05 was considered indicative of statistical significance.

## Data Availability

All data generated during this study are included in this article.
